# Evaluation of the effectiveness of a postnatal support education program for husbands in promotion of their primiparous wives’ perceived social support: a randomized controlled trial

**DOI:** 10.1186/s12905-023-02270-x

**Published:** 2023-03-28

**Authors:** Zahra Abbaspoor, Foruzan Sharifipour, Mojgan Javadnoori, Zahra Behboodi Moghadam, Bahman Cheraghian, Mahin Najafian

**Affiliations:** 1 Department of Midwifery, Reproductive Health Promotion Research Center, Ahvaz Jundishapur University of Medical Sciences, Ahvaz, Iran; 2 Department of Midwifery, School of Nursing and Midwifery, Kermanshah University of Medical Sciences, Kermanshah, Iran; 3 Department of Midwifery, Reproductive Health Promotion Research Center, Ahvaz Jundishapur University of Medical Sciences, Ahvaz, Iran; 4 School of Nursing and Midwifery, Tehran University of Medical Sciences, Tehran, Iran; 5 Department of Biostatistics and Epidemiology, School of Public Health, Alimentary Tract Research Center, Clinical Sciences Research Institute, Ahvaz Jundishapur University of Medical Sciences, Ahvaz, Iran; 6 Department of Obstetrics and Gynecology, School of Medicine, Fertility Infertility and Perinatology Research Center, Ahvaz Jundishapur University of Medical Sciences, Ahvaz, Iran

**Keywords:** Social support, Primiparous women, Educational program, Postpartum care

## Abstract

**Background:**

Primiparous women experience various challenges if not provided with social support in the early postpartum period. Support in form of postpartum education programs is needed to improve mental well-being in primiparous women. The aim of this study was to determine the effect of a postnatal supportive education program for husbands on the perceived social support (primary outcome), and stress and maternal self-efficacy (secondary outcome) of their primiparous wives.

**Methods:**

This randomized clinical trial was performed on pregnant women referring to healthcare centers for routine care from September to November 2021 in Kermanshah, Iran. One hundred pregnant women were randomly divided in to intervention and control groups. Four 45–90 min online training sessions were held weekly for the husbands of the intervention group. The primiparous women completed the Postpartum Partner Support Scale, Perceived Stress Scale, and Postpartum Parental Expectations Survey before (third day after delivery, immediately and one month after completing the intervention. Data were analyzed using Fisher's exact test, Chi-square test, independent t-test, and repeated measures analysis of variance in SPSS version 24, and *p* < 0.05 was considered statistically significant.

**Results:**

In the control and intervention groups before the intervention, socio-demographic characteristics (*P* > 0.05), the mean scores of perceived social support (*P* = 0.11), maternal self-efficacy (*p* = 0.37) and perceived stress (*p* = 0.19) were not statistically significant. However, in the intervention group compared to the control group the mean scores of perceived social support (79.42 ± 7.17 vs. 37.26 ± 7.99, *P* < 0.001), maternal self-efficacy (186.22 ± 39.53 vs. 106.3 ± 32.88, *P* < 0.001) and perceived stress (16.36 ± 6.65 vs. 43.3 ± 7.39, *P* < 0.001) immediately after the intervention and the mean scores of perceived social support (84.4 ± 5.91 vs. 37.14 ± 6.63, *P* < 0.001), maternal self-efficacy (191.24 ± 38.92 vs. 112.34 ± 37.12, *P* < 0.001) and perceived stress (13.98 ± 4.84 vs. 39.06 ± 7.25, *P* < 0.001) one month after the intervention changed significantly.

**Conclusion:**

The postpartum supportive education program for husbands was effective in promoting social support for primiparous women. Thus it can be introduced as routine care in the postpartum period.

**Trial registration:**

Clinical trial registration Iranian Registry of Clinical Trials; https://en.irct.ir/user/trial/56451/view (IRCT20160427027633N8), registered (15/06/2021).

**Supplementary Information:**

The online version contains supplementary material available at 10.1186/s12905-023-02270-x.

## Introduction

The early postpartum period is an exciting and joyful time for many parents, yet it is a stressful transient period during which most primiparous women face a variety of physical and emotional challenges, including fatigue and difficult responsibilities of caring for the baby and profound changes in the couple's roles and responsibilities [1].

The postpartum period is characterized by the vulnerability of primiparous women to stress. Postpartum stress not only impairs the health of primiparous women but also reduces their self-esteem, impedes adaptation to the role of mother, and impairs maternal bond with the baby [2]. Common stressors of the postpartum period include breastfeeding problems, sleep deprivation, fatigue, responsibility for caring for the baby, hormonal changes, and lack of social support [3].

Maternal self-efficacy refers to the mother’s belief in her abilities as an efficient mother and is greatly influenced by the sense of motherhood, self-confidence and perception of empowerment [4]. High self-efficacy is associated with positive thoughts, higher self-esteem, better adjustment, and more positive emotions [5]. Various studies have shown a negative relationship between maternal self-efficacy and maternal stress and postpartum depression [1, 6]. Some studies have shown that education during pregnancy is insufficient for the woman to play the maternal role and has no effect on maternal skills [7, 8].

Barnard (1994) emphasizes the importance of social support in the first year after childbirth [9]. Perceived social support refers to the amount of love, companionship, care, respect, attention, and help a person receives from others, such as family members, friends, etc. [10] There are three main types of social support: 1. Emotional support, which means having someone available to rely on and trust when needed. 2- Information support, which indicates receiving useful information (including guidelines, suggestions, advice, and feedback) from others to adapt to personal problems, and 3- Instrumental support, which is the material, objective and actual help received by a person from others [11]. A previous study found that pregnant women who received high social support from their husbands during pregnancy experienced less stress [12].

The results of a randomized controlled trial demonstrated the effectiveness of the postnatal psychoeducation program in improving maternal parental self-efficacy, social support and reducing postnatal depression of primiparous women [13]. Also, various studies have shown that social support is negatively correlated with the risk of postnatal depression [14, 15]. Hence, providing strong social support to primiparous women can potentially prevent them from developing postnatal depression (PND). Supportive interventions can be facilitated by providing social support in the form of education and maintaining the continuity of cares postnatal [16]. Shorey et al. (2017) demonstrated that the educational programs based on mobile applications are effective in improving parental self-efficacy and social support, recommending it to be introduced and performed by nurses in routine care [17]. However, the provision of supportive interventions is not widely practiced during the postnatal period as the focus has been mainly limited to pregnancy and childbirth [18]. Primiparous women often cite their family members, especially their husbands, as the main source of social support [1]. According to the opinion of most women, a woman's husband is the most important and closest person who can support her in coping with the problems by carefully understanding her sensitive psychological and physical condition [19]. Fletcher et al. showed that fathers’ participation in classes before delivery was effective in their supportive role [20]. The results of various studies have shown that fathers’ participation in pregnancy and childbirth care has positive consequences for the mother and baby, including reduced maternal stress, pregnancy complications (e.g., preterm delivery) and fear of childbirth, proper weight gain of premature infants, and successful breastfeeding. This is even true with long-term effects such as improved language learning and children’s academic achievement [21, 22]. Various studies recommended husband counseling and education as effective interventions to increase social support for pregnant and postpartum women [23–26].

In some Western countries, mothers and their infants are discharged early to facilitate family-centered postnatal care and encourage a sense of parental involvement. [27] In Iran, the average length of hospital stay of mothers after childbirth is 1 to 2 days [28]. Due to the mother’s short stay in the hospital, there is not sufficient time for educational interventions [17]. Moreover, the period immediately after delivery is not a good opportunity for education due to the discomfort that mothers experience during this period [29]. Parents are often overwhelmed by the amount of information they are given during their short hospital stay. Some complain that they have difficulty retaining this information after discharge and prefer to receive the information continuously after discharge early in the postpartum period [27]. To address these issues, healthcare providers need to develop the knowledge and tools needed to improve the quality of postnatal care that parents expect [30].

Given the importance of primiparous women’s mental health in facilitating adaptation to changes and new roles in the during the postpartum period, many husbands have a vital and decisive role in supporting their wives during this period. However, there is no sufficient time to provide all the necessary information in a short period of hospitalization, and according to a conducted survey, no study was found on the effectiveness of a postnatal supportive education program for husbands on perceived social support, stress and maternal self-efficacy of their primiparous wives. Therefore, the present study was conducted to determine the effect of a postnatal supportive education program for husbands on perceived social support (primary outcome), and stress and maternal self-efficacy (secondary outcome) of their primiparous wives.

### Hypothesis of study

Education to the husbands of primiparous women will lead increased perceived social support and maternal self-efficacy and reduced stress of these women in the postpartum period.

### Primary aim

Determine the effect of a postnatal supportive education program for husbands on perceived social support of their primiparous wives.

### Secondary aim

Determine the effect of a postnatal supportive education program for husbands on stress and maternal self-efficacy of their primiparous wives.

## Methods

### Study design and participants

**Fig. 1:**
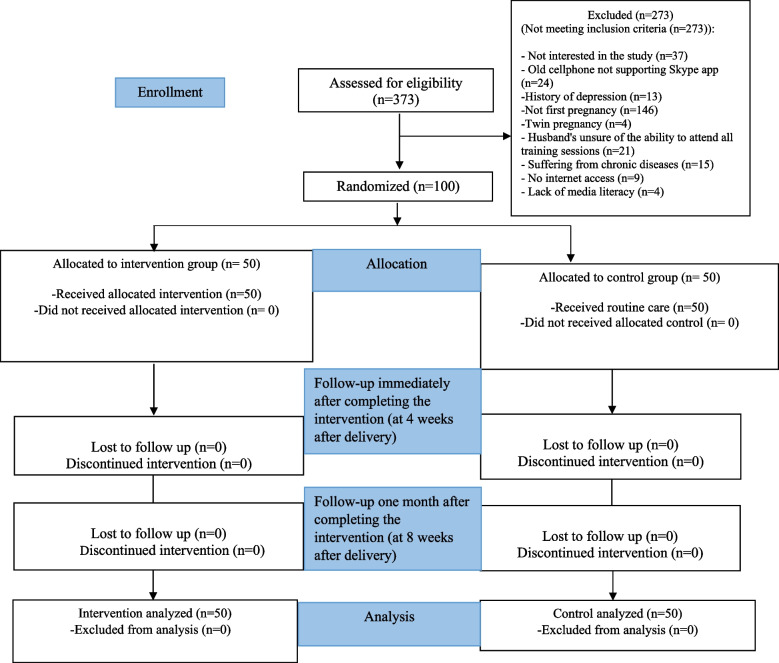
CONSORT flowchart of the study

This randomized, controlled clinical trial study using two parallel groups was performed on 100 pregnant women referring to health centers in Kermanshah, Iran from September to November 2021. Figure 1 shows the CONSORT flowchart of the study. Women meeting the following criteria were eligible to participate in the study: first pregnancy, singleton term pregnancy, willingness to participate in the study, married and living with her husband, husband’s willingness to participate in the study, media literacy (familiarity with how the Skype app works), having a smartphone, knowing how to install and work with Skype, access to the Internet, and being available at least within the next 8 weeks. Exclusion criteria included: being husband's unable or unsure of the ability to attend all training sessions, presence of cardiovascular disease, high blood pressure, liver disease, diabetes or other chronic diseases (as reported by the woman), neuropsychiatric diseases, having recent calamities for the participant’s first-degree relatives (as reported by the woman) (such as death or incurable disease), and attending similar training or counseling classes by the husband before the study.

### Sample size calculation

The sample size was calculated based on the main variable of the study (social support) according to Khanzadeh and Moghaddam Tabrizi [31] using the following formula with type I error (α) set as two-sided 5% (Z1 − α/2 = 1.96), type II error (β) set as 20% (Z1 − β = 0.85) and power of 80%. The sample size for both groups was obtained 86 subjects and considering a 10% attrition rate, the final sample size was calculated to be 96 for both groups (48 each). Based on the opinion of the research team, we rounded the number 96 to 100 (50 each group).

$$n=\;\frac{\left(z_{1-\frac\alpha2}+_{1-\beta}\right)^2+\left(SD_1^2+SD_2^2\right)}{\left(U_1-U_2\right)^{\wedge2}}$$

μ1: mean difference of the social support in the intervention group (11.25), μ2: mean difference of the social support in the control group (4.95), SD1: standard deviation of the social support before intervention (14.21), and SD2: standard deviation of the social support after intervention ($10.00$).

### Sampling and random allocation method

The present study was approved by the committee of ethics of Ahvaz Jundishapur University of Medical Sciences (IR.AJUMS.REC.1399.401) and was registered in Iranian Registry of Clinical Trials (IRCT20160427027633N8). First, According to the information of Kermanshah health center, Kermanshah city was divided into 4 geographical regions (or 4 classes) based on socio-economic status of the residents. These four regions included 76 healthcare centers, and from the healthcare centers of each region, one was selected by simple random method (drawing lots). These centers were Taleghani, Hafezieh, Shahid Souri, and Haj Daei, from which 28, 34, 22, and 16 women were selected by convenience sampling method (based on the share of each center). After making the necessary arrangements and obtaining a permit, the researcher went to these centers and selected eligible women. In this way, by referring to the Sib system, information about the women who were covered by the center and met the study entry criteria was extracted and they were contacted by phone to come to the health center to participate in the study. The study aims and method were explained to pregnant women and their husbands in a face-to-face meeting and informed written consent was gained from them (pregnant women and their husbands). The telephone numbers of the women and their husbands were taken, and the researcher gave her telephone number to them, and they were advised to inform the researcher when the mother gave birth to the baby. After the women completed the questionnaires online on the third day after delivery, the educational content was offered to the intervention group. It should be noted that the membership of the husbands in the virtual (Skype) group was monitored by checking whether or not they had installed Skype on their cellphones, and in case they had not, it was installed on their cellphones. Necessary training on how to use Skype was given to them.

The women were randomly divided into 6 blocks with an allocation ratio of 1:1 to the intervention and control groups. Blocked randomization was performed by a person who was not involved in data collection, and in order to conceal random allocation, opaque sealed packets in random sequence were used. To this aim, first, a random sequence was created by a table of random numbers and recorded on a card and the cards were placed in packets according to the random sequence. Packets were opened in the order of woman entry in the study and they were allocated into either the intervention or control group. Data analysis was performed by a statistician who was unaware of the nature of the codes. Due to the nature of the intervention, it was not possible to blind neither the participants nor the researchers.

### Intervention

Training was performed for intervention group in Skype. While the control group received only routine care. Training involved 4 online sessions of 45 to 90 min in Skype, held weekly (one session per week) by the researcher. Due to the different nature of education on natural childbirth and cesarean section, the women in the intervention group were divided according to the type of delivery (32 women who were supposed to have a normal delivery were in 4 groups of 8, and 18 women who were going to deliver their baby by a cesarean section were in 3 groups of 6). Topics of training sessions included: first session: the importance of maternal health in the postpartum period and the importance of social support for the mother in this period, second session: anatomical and physiological changes in the postpartum period, the impact of these changes on the mothers mental state, and the way she should be cared for, third session: baby care, the principles of breastfeeding and how to deal with its challenges, fourth session: the father’s responsibilities in the postpartum period and his supportive role in helping mothers adapt to changes (Interventional group educational content sessions are available in Additional file 1: Table 1).

**Table 1:** Socio-demographic characteristics of the intervention and control groups

Variables	Intervention *n* = 40	Control *n* = 40	*P* value
	**Mean ± SD** **Or N (%)**	**Mean ± SD** **Or N (%)**	
**Age (years)**	29.3 ± 6.16	28.14 ± 5.98	*0.34
**Husband’s age (years)**	35.08 ± 5.58	33.56 ± 5.13	*0.16
**Age gap with husband (years)**	5.56 ± 2.67	5.42 ± 2.73	*0.79
**Weight at birth (grams)**	30.30 ± 180.98	30.76 ± 171.2	*0.19
**Education**			***0.6
Primary high school	6(12)	5(10)	
Secondary high school(diploma)	8(16)	12(24)	
University	36(72)	33(66)	
**Occupation**			**0.7
Employed	11(22)	14(28)	
Housekeeper	27(54)	21(42)	
Student	10(20)	12(24)	
Other	2(4)	3(6)	
**Husband’s education**			**0.22
Primary high school	6(12)	3(6)	
Secondary high school(diploma)	19(20)	17(34)	
University	34(68)	30(60)	
**Husband’s occupation**			**0.84
Employed	32(64)	28(56)	
Jobless	5(10)	6(12)	
Worker	12(24)	14(28)	
Other	1(2)	2(2)	
**Economic status**			***0.4
Weak	4 (8)	6(12)	
Medium	39(78)	33(66)	
Good	7(14)	11(22)	
**Husband’s disease**			***0.18
No	44(88)	39(78)	
Yes	6(12)	11(22)	
**Delivery method**			**0.4
Vaginal	30(60)	34(68)	
Cesarean section	18(36)	16(32)	
Vacuum delivery	2(4)	0(0)	
**Type of baby feeding**			***0.55
Breast milk	31(64)	36(72)	
Milk powder	4(8)	2(4)	
Both	15(30)	12(24)	
**Breastfeeding problems**			***0.42
Breast congestion	5(10)	8(16)	
Nipple sores	8(16)	11(22)	
No problem	37(74)	31(62)	
**Pregnancy status**			***0.53
Planned pregnancy	45(90)	43(86)	
Unplanned pregnancy	5(10)	7(14)	
**Baby’s gender**			***0.23
Girl	21(42)	27(54)	
Boy	29(58)	23(46)	
**Satisfaction of the baby’s gender**			
Yes	50(100)	50(100)	
No	0(0)	0(0)	

*Independent t-test, ** Fisher’s exact test, *** chi-square test

The educational content was prepared by referring to the available library resources and with the guidance of supervisors and consultants and also based on the instructions and guidelines of the Iranian Ministry of Health. The validity of the educational content was evaluated qualitatively and approved by 10 relevant experts and faculty members related to the subject under study, including four reproductive health specialists, one midwifery specialists, three gynecologists and two psychologists. Each week after the review session, the content was presented in text, video and audio formats to the intervention group until the next session. Meetings were held upon agreement of the husbands. Each week, the researcher reminded the audience of the time of the training sessions by phone and text messages. In addition, the intervention group provided access to the researcher to ask questions via WhatsApp. Immediately and one month after completing the intervention (i.e., the fourth and eighth weeks after delivery), the questionnaire was completed by primiparous women online. In the end, in order to observe the ethical considerations, the educational materials were provided to the control group.

### Study outcomes

In this study, the study outcomes (primary outcome: social support and secondary outcome: maternal self-efficacy and stress) were measured at three time intervals: baseline or before intervention (third day after delivery), immediately after the intervention (fourth week after delivery), and one month after the intervention (eighth week after delivery). The outcome measures were completed by primiparous women online.

### Data collection tools

Data collection tools included socio-demographic questionnaire, Postpartum Partner Support Scale, Perceived Stress Scale, and Postpartum Parental Expectations Survey.

#### Socio-demographic questionnaire

This questionnaire includes questions related to age, husband’s age, age gap with husband, educational of the woman and her husband, employment status of the woman and her husband, economic status, husband’s disease, weight at birth, type of baby feeding, breastfeeding problems, delivery method, pregnancy status, and baby’s gender and parental satisfaction with.

### Primary outcome

#### Postpartum Partner Support Scale (PPSS)

This scale was developed in 2017 by Dennis et al. This questionnaire has a four-point Likert scale (from *strongly disagree*: 1 to *strongly disagree*: 4). Scores of this questionnaire range from 20 to 100, and higher scores indicate receiving higher husband support in the postpartum period [32]. The reliability of this questionnaire in the original version was reported by obtaining a Cronbach’s alpha coefficient of 0.96 [23]. In Iran, Moghaddam Tabrizi and Khanzadeh (2020) reported a Cronbach’s alpha of 0.96, an internal stability index was 0.7, and alpha and reliability of 0.98. Also, the content validity index (CVI) and the content validity ratio (CVR) of this scale were 70% and 81%, respectively, which are both acceptable [31].

### Secondary outcome

#### Postpartum Parental Expectations Survey (PPES)

This scale designed by Reese in 1992, was used to measure maternal self-efficacy. This questionnaire has 25 affirmatively worded items scored based on a 10-point Likert scale (from *I can't:* 1 to *I definitely can*: 10). The minimum score of this questionnaire is 25 and the maximum is 250, with scores less than 25 indicating lack of self-efficacy of the mother and those higher indicating more self-efficacy of the mother. The validity and reliability of this questionnaire was first confirmed by Reese in 1992 [33]. The validity and reliability of this questionnaire in Iran was confirmed by Jafarnejad et al. in 2014 by obtaining a Cronbach's alpha coefficient of 0.87, a content validity index of 0.79, and a content validity ratio of 0.62 [34].

#### Perceived Stress Scale (PSS)

Perceived Stress Scale was developed in 1983 by Cohen et al. to measure general perceived stress over the past month. Thoughts and feelings about stressful events, as well as controlling, overcoming, coping with stress. In the present study, the 14-item version was used which is answered based on a 5-point Likert scale (from *Never:* 0 to *Most of the time*: 4). Total Scores range from zero to 56, with higher scores indicating more perceived stress. The reliability of this questionnaire in the original version using Cronbach’s alpha coefficient method has been reported between 0.84 and 0.86, and its correlations with the constructs measured are also high (0.52 to 0.76) [35]. In Iran, the reliability of this questionnaire was calculated by Bastani et al. using the internal consistency method and obtaining a Cronbach’s alpha coefficient of 0.74 [36]. Dolatian et al. (2014) reported a content validity index of 95% and a content validity ratio of 90% for this questionnaire [37].

### Data analysis

The collected data were analyzed using descriptive statistics (frequency, percentage, mean and standard deviation) and inferential statistics using SPSS version 24. *P* < 0.05 was considered statistically significant. Kolmogorov–Smirnov test was used to evaluate the normality of quantitative data. Fisher’s exact test, Chi-square test, and independent t-test were used to examine the demographic characteristics. Independent t-test and analysis of variance with repeated measures were used to compare the mean scores of social support as well as perceived stress, and maternal self-efficacy in groups at each time interval and over time, respectively.

## Results

In this study, of the 373 primiparous women referring to health centers who were initially enrolled, 273 were excluded from the study due to not meeting inclusion criteria. No attrition was observed in both groups during the study period, and all participants (50 in the intervention group and 50 in the control group) were analyzed at two intervals (immediately and one month after the intervention) after the follow-up period (Fig. 1). The demographic characteristics of the participants are shown in Table 1. The mean age of women and their husbands were 29.3 ± 6.16 and 35.08 ± 5.58 in the intervention group and 28.14 ± 5.98 and 33.56 ± 5.13 in the control group, respectively. There was no significant difference between the two groups in terms of demographic characteristics.

**Fig. 2:**
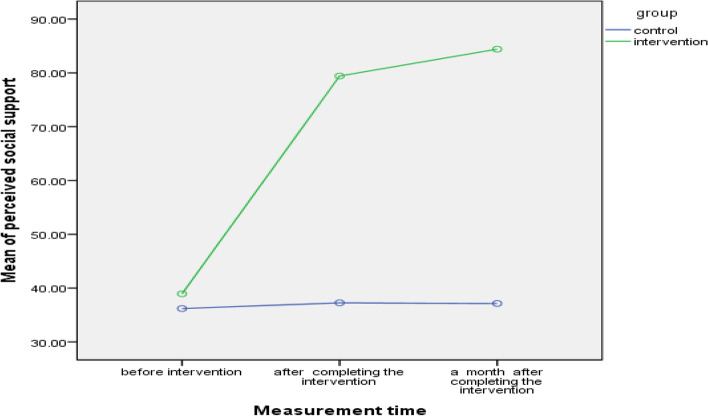
The mean scores of perceived social support in the intervention and control groups across the three measurement times

**Fig. 3:**
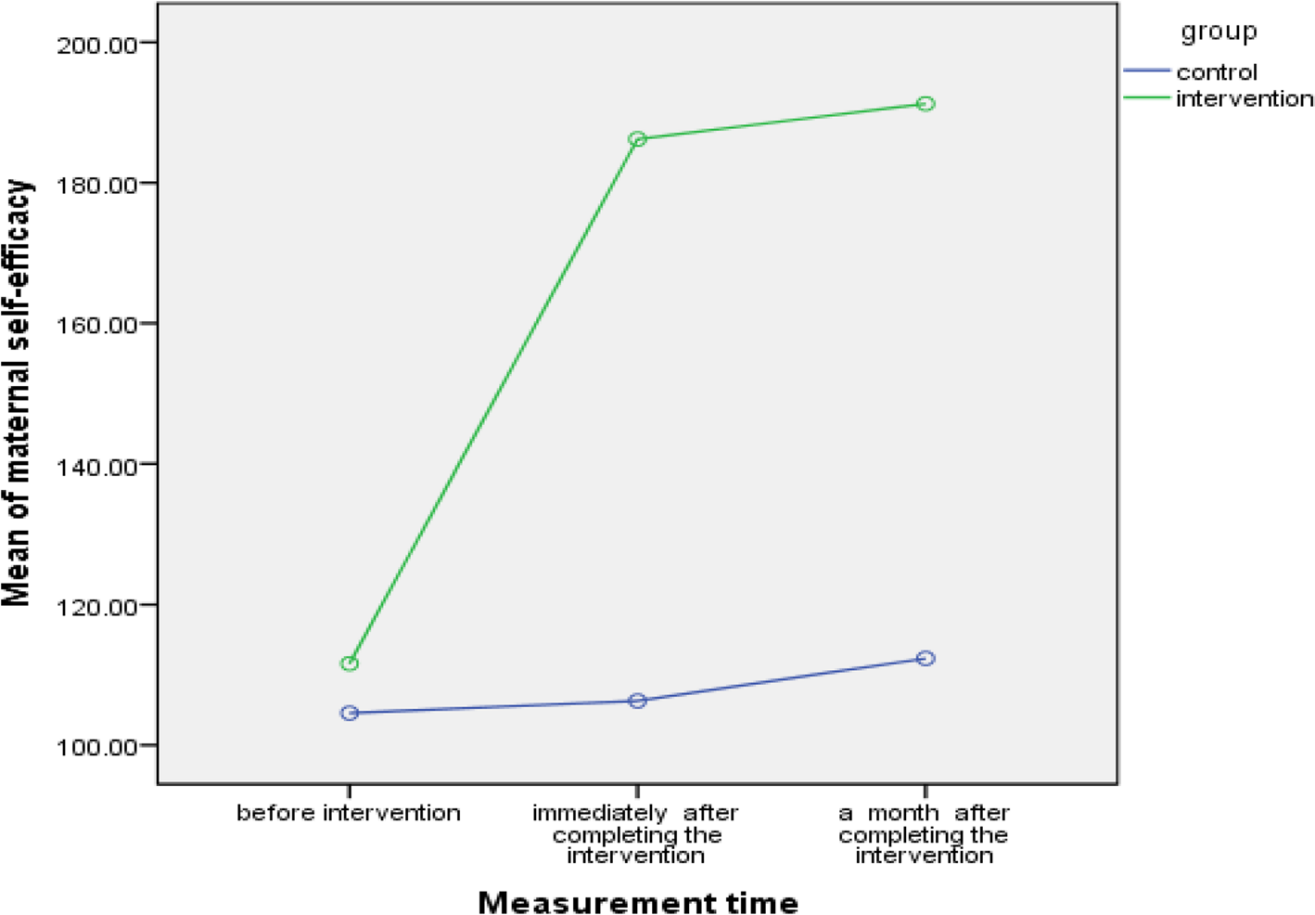
The mean scores of Maternal self-efficacy in the intervention and control groups across the three measurement times

**Fig. 4:**
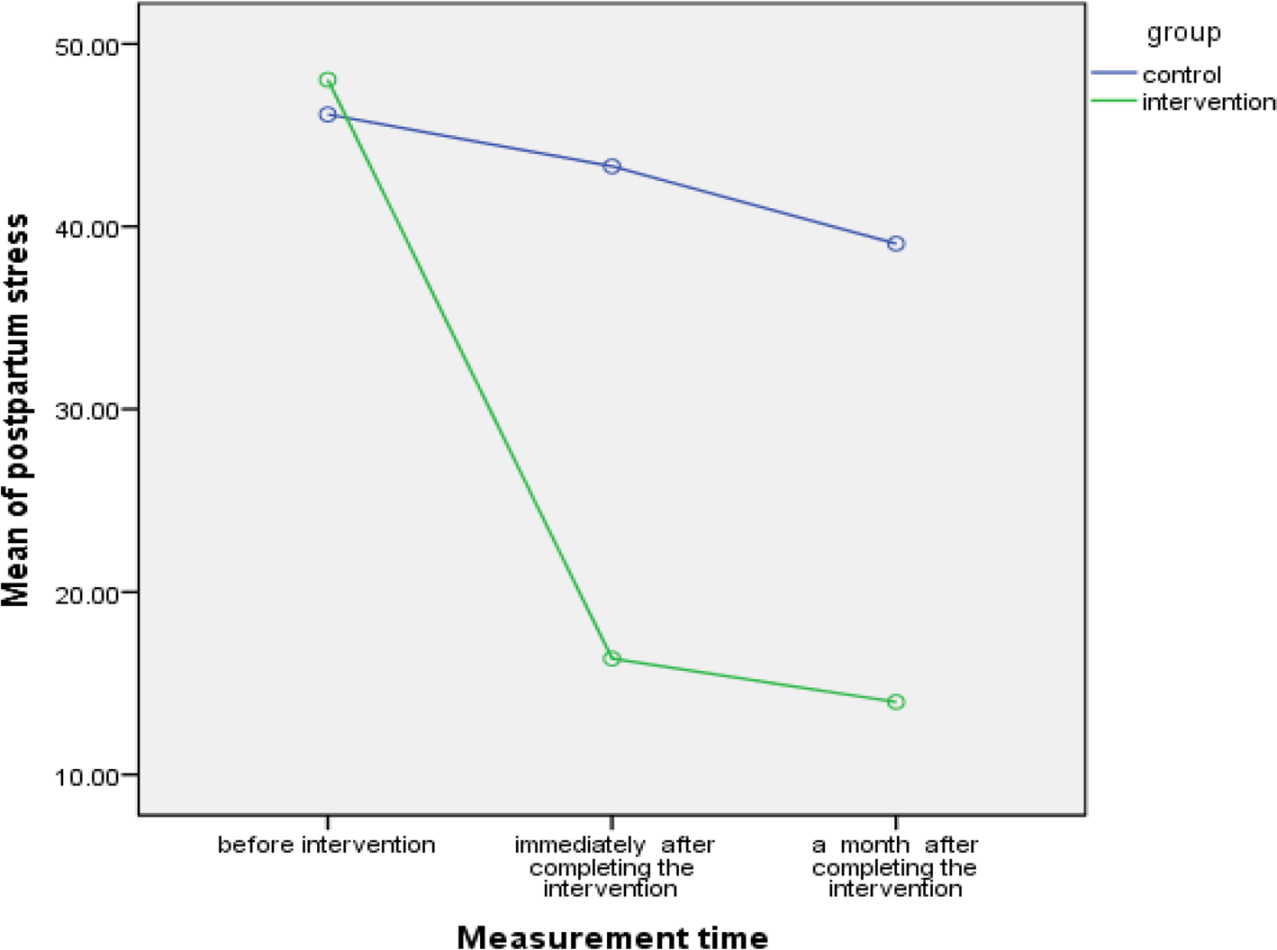
The mean scores of Perceived postpartum stress in the intervention and control groups across the three measurement times

Based on independent t-test, the mean scores of perceived social support (*P* = 0.11), maternal self-efficacy (*p* = 0.37) and perceived stress (*p* = 0.19) before intervention were not significantly different in the two groups. In the intervention group, the mean scores of perceived social support, self-efficacy and perceived stress of primiparous women immediately and one month after completing the intervention were significantly different from those in the control group (*P* < 0.001). Analysis of variance with repeated measures showed that the perceived social support (mean difference = 30.72; 95% confidence interval = 28.61 to 32.82; *p* < 0.001), maternal self-efficacy (mean difference = 55.29; 95% confidence interval = 41.85 to 68.73; *p* < 0.001) and perceived stress (mean difference = -16.7; 95% confidence interval = -18.71 to -14.70; *p* < 0.001) of primiparous women in the intervention group changed significantly over time, and the interaction analysis showed that there was a significant cross impact between group and time (*P* < 0.001) (Table 2). As shown in Figs. 2, 3 and 4, the mean scores of perceived social support and maternal self-efficacy in the intervention group increased over time while the mean scores of perceived stress decreased over time.

**Table 2:** Comparisons of the mean scores social support, self-efficacy and perceived stress at three measurement time points (before intervention, immediately and a month after completing the intervention) in the studied groups

Variables	Intervention (*n* = 50) Mean ± SD	Control *n* = 50 Mean ± SD	Mean differencea (CI = 95%)a	*P* value*			
**Perceived social support (20–100)**							
Before intervention	8.8 ± 38.94	8.43 ± 36.32	-2.74(-6.17–0.69)	*P* = 0.11			
Immediately after completing the intervention	7.17 ± 79.42	37.26 ± 7.99	-42.16(-45.18- -39.15)	*P* < 0.001			
A month after completing the intervention	5.92 ± 84.40	6.64 ± 37.14	-47.26(-49.76- -44.77)	*P* < 0.001			
*P* value**	*P* < 0.001	*P* = 0.62	30.72(28.61- 32.82) *P* < 0.001			Time effect *P* < 0.001	Group*Time effect *P* < 0.001
**Maternal self-efficacy (25–250)**							
Before intervention	111.60 ± 42.43	104.56 ± 37.04	-7.04(-22.85–8.77)		P = 0.37		
Immediately after completing the intervention	186.22 ± 39.53	106.3 ± 032.88	-79.92(-94.35- -65.49)		P < 0.001		
A month after completing the intervention	191.24 ± 38.92	112.34 ± 37.12	-78.90(-93.99- -63.80)		P < 0.001		
*P* value**	*P* < 0.001	*P* = 0.06	55.29(41.85- 68.73) *P* < 0.001			Time effect *P* < 0.001	Group*Time effect *P* < 0.001
**Perceived postpartum stress (0–56)**							
Before intervention	48.04 ± 7.06	46.14 ± 7.37	-1.90(-4.76- 0.97)		*P* = 0.19		
Immediately after completing the intervention	16.36 ± 6.65	43.3 ± 07.39	26.94(24.15–29.73)		*P* < 0.001		
A month after completing the intervention	13.99 ± 4.84	39.06 ± 7.25	25.08(22.63–27.52)		*P* < 0.001		
*P* value**	*P* < 0.001	*P* = 0.10	-16.7(-18.71- -14.70) *P* < 0.001			Time effect *P* < 0.001	Group*Time effect *P* < 0.001

^*^Independent t-test; **Repeated Measures ANOVA

## Discussion

The results of this study showed that the postnatal supportive education program for husbands enhanced the understanding of social support for their primiparous women in the fourth and eighth weeks after delivery. It can be argued that providing education and consultation services to husbands in order to increase their awareness about the characteristics and issues of women in the postpartum period is one of the tools for promoting husbands’ social support to their wives, which has a positive effect on the couple’s knowledge, attitude and practice with respect to postpartum health [31]. Various studies have introduced the husband as the most important source of support in stressful life situations of women [38, 39].

To the best of our knowledge, no study has yet been conducted on the effect of husband education or counseling on social support, maternal self-efficacy, and stress in postpartum women. However, previous studies have shown the overall impact of participation and counseling of pregnant women's husbands, primiparous women and couples in this respect. Mohammadpour et al. (2020), for instance, conducted a randomized clinical trial to determine the effect of husband counseling on social support perceived by their pregnant wives. Their intervention involved four 60-min sessions of group counseling (each group consisting of 7 to 10 people) once a week for four consecutive weeks. The results showed that the mean score of social support 4 weeks after the intervention in the intervention group increased significantly compared to the control group [23]. Another study in Singapore conducted by Shorey et al. (2017) examined the effectiveness of a postpartum psychiatric educational mobile application called "Home—but not Alone" in improving parenting outcomes. Parents in the intervention group received a training program in addition to routine care while the control group received only routine care. The results showed that parental self-efficacy and social support significantly improved four weeks after delivery in the intervention group compared to the control group [17]. The results of studies of Turan et al. and Sahip et al. showed that husbands’ education led to improvement their more appropriate supportive behaviors with women during pregnancy [25, 26]. The results of the present study are in line with those mentioned above, which indicates the positive effect of husband education or counseling on mothers’ social support. Therefore, it is necessary to increase the awareness of husbands about their positive role in the postpartum period and their participation in promoting the health of mother and baby. In fact, in the present study, online education through social networking provided a virtual community for husbands, and facilitated social support between members of this community through sharing personal information and experiences, and at the same time, and created the opportunity of making friends with other men in this community.

The results of the present study showed that the postnatal supportive education program for husbands of primiparous women promoted maternal self-efficacy in these women in the fourth and eighth weeks after delivery. It can be argued that educating men to promote support for their primiparous wives can play an effective role in reinforcing the mother’s abilities and her interpretation of the competence she has to play the role of a mother, thus increasing her self-efficacy [40]. The results of previous studies confirm our findings with respect to the positive effect of educational intervention on the promotion of maternal self-efficacy, and this was achieved in our study 4 and 8 weeks after delivery [34, 41]. In line with the present study, the results of a randomized clinical trial showed the significant effect of a postpartum psychological education program on increasing maternal self-efficacy and social support and reducing postpartum depression among primiparous women in their 6th and 12th weeks of postpartum. The final results of this trial indicated that the postpartum psychological education program is effective in improving maternal outcomes [13]. The main difference between this study and the present study was that in the former the training program involved a face-to-face meeting and presentation of a booklet for primiparous women, while in our study the training program included 4 online training sessions for husbands. In fact, the training sessions in our study were for husbands only. In these sessions, the need for spouse support in the postpartum period was taught for fathers and we assessed the effects of education on their primiparous women indirectly.

Salonen et al. (2010) investigated the effect of an online educational intervention on increasing maternal satisfaction and self-efficacy. Mothers in the intervention group had access to an educational website offering infant care training content from the 20th week of pregnancy whereas the control group received only the routine training. No statistically significant difference was observed between the two groups 6 to 8 weeks after delivery in terms of self-efficacy and parental satisfaction compared to before the intervention [42]. This finding is contrary to the results of the present study. This discrepancy can be attributed to the fact that participants in their study included a combination of primiparous and multiparous women with full-term or pre-term pregnancies as well as normal or abnormal birth weight. In addition, a number of women in both groups in their study had a depression score higher than 10, which was not the case in the present study.

In the present study, participants had access to information through education. This useful information helped the men to acquire the right knowledge independently and, by providing support to the mother, they managed to perform the challenging parenting tasks and increase the mother’s self-efficacy. The helpful feedback given to the husbands served as verbal encouragement, thus allaying their concerns and increasing their self-confidence and motivation to promote maternal self-efficacy.

The results of the present study showed that the postnatal supportive education program for husbands of primiparous women reduced the perceived stress in these women in the fourth and eighth weeks after delivery. This can be attributed to the fact that educating husbands on providing emotional, informational, and instrumental support to the mother reduces her physical and psychological stress. Therefore, among the available support sources, the support provided by sexual partners is very important [43]. This finding is consistent with the results of studies by Cohen et al. (2014) and Dafie et al. (2021) where providing counseling services to couples based on cognitive-behavioral methods during pregnancy has been found to exert a positive effect on reducing stress and depression. In this method, perinatal stress and depression is reduced and post-natal mental health is promoted by enhancing the couples’ understanding about pregnancy and its changes, minimizing negative behaviors and attitudes, increasing emotional support, and promoting empathetic communication during pregnancy. Given the relationship between mental health during pregnancy and postpartum mental health, the use of educational and counseling methods in care centers to promote maternal mental health is strongly recommended [44, 45].

Also, study of Alio et al. (2013) showed that the primary benefits of male partner involved during pregnancy were the reduction of maternal stress levels and the encouragement of positive maternal behaviors [46]. In another study, the results showed that 4 sessions of consultation with fathers had no effect on reducing stress in pregnant mothers [23], which is contrary to the results of the present study. It seems that the difference is attributable to the type of intervention, educational content, number and type of participants, follow-up period, or different assessment tools. Research has shown that educational information provided by health care providers during pregnancy is rarely adhered to by mothers themselves during the postpartum period [47], indicating the mothers’ need for support to cope with stress correctly, which explains the results of the present study.

One of the limitations of this study was that women in this study had no neuropsychiatric diseases. This may affect the generalizability of the results to women who are depressed. The second limitation was that data collection was based solely on participants’ own reports, and the researchers did not use other sources of data collection such as observation. The third limitation was that due to the nature of the intervention, the researchers were not blind to data collection after the intervention. Also, no data was collected from husbands. The last limitation was the cultural component. This prevents generalization to other realities. There is no homosexual culture in Iran, and families made up of a man and a woman and who are married. Other family models (single parent, mixed families, not married, formed by people of the same sex), which are not considered here and could be considered in other studies. These limitations notwithstanding, this study is worthwhile considering the following: Random allocation, concealment of allocation, random selection of women from health centers in 4 districts of the city, long-term follow-up, providing telephone numbers to answer participants’ questions, and providing educational content to the control group at the end of the study. Also, the intervention was designed around the planned delivery method. Therefore, we suggest that a similar study be done in women with a history of depression or suffering from that with more sophisticated statistical analysis.

## Conclusion

This study demonstrated the effectiveness of a postnatal supportive education program for husbands (male partners) in improving perceived social support, maternal self-efficacy, and stress in primiparous women. Therefore, policymakers in postpartum care are advised to consider the participation of husbands in the postpartum care process and to devise plans to increase the awareness of husbands and their role in promoting maternal and infant health. Also, to confirm the results of this study, a randomized controlled trial with a larger sample size from the whole population is suggested.

## Supplementary Information

**Additional file 1: Table 1.** Intervention group educational content sessions.

## Data Availability

The data that support the findings of this study are available from the corresponding author upon reasonable request.

## Abbreviations

PND: Postnatal Depression
PSS: Perceived Stress Scale
PPES: Postpartum Parental Expectations Survey
PPSS: Postpartum Partner Support Scale
CVI: Content Validity Index
CVR: Content Validity Ratio
