# Supplementary material for: Evaluation of the effectiveness of a postnatal support education program for husbands in promotion of their primiparous wives’ perceived social support: a randomized controlled trial
Source: BMC Womens Health. 2023 Mar 28;23:139. doi: 10.1186/s12905-023-02270-x (PMC10045875; doi:10.1186/s12905-023-02270-x)
Supplement: Supplementary file 1 — Additional file 1: Table 1. Intervention group educational content sessions. [file 12905_2023_2270_MOESM1_ESM.docx]

Table 1: Intervention group educational content sessions

|  | **Main content** | **Educational content** | **Training material** | **Educational method** |
| --- | --- | --- | --- | --- |
| **1** | Importance of maternal health in the postpartum period and the importance of social support for the mother in this period | - Introduction and acquaintance of members - Initial evaluation - Explain the goals - General explanation of the details of the 4 sessions - Emphasis on full participation in the training course - Definition of puerperium - Adaptation to parenthood - Definition of social support - Types of social support - Social support and health - The importance of understanding the support of the spouse - Summary of the meeting - Answer the questions | Virtual webinar and group discussion | - PowerPoint slides - Video files - Text messages - Audio file |
| **2** | Anatomical and physiological changes in the postpartum period, the impact of these changes on the mothers mental state, and the way she should be cared for | - Review the previous session summary - Changes in the mother's body system during this period and related issues - Common complaints in this period and how to deal with it - Symptoms of maternal danger in the postpartum period - Maternal psychological changes in the postpartum period and how to deal with it - Necessary recommendations to prevent psychological changes in the mother - Caring for the mother after childbirth after discharge from the hospital and providing the necessary recommendations - Recommendations for safe sexual activity in the first weeks - How to breastfeeding - Summary of the meeting - Answer the questions | Virtual webinar and group discussion | - PowerPoint slides - Video files - Text messages - Audio file |
| **3** | Baby care, the principles of breastfeeding and how to deal with its challenges | - Review the previous session summary - Characteristics of a healthy baby - Periodic examinations and screening of the baby after discharge - Necessary recommendations to maintain the safety of the baby - Vital signs of the baby - Sleeping position of the baby - Causes of baby crying - Baby bathing - Signs of hunger and satiety in infants - Caring for a circumcised baby boy - Danger symptoms in infants - Common problems of infancy - Necessary recommendations in baby care - Benefits of breastfeeding - Prohibitions on breastfeeding - Effective factors in strengthening breastfeeding - Proper breastfeeding technique - Common false beliefs about breastfeeding - Definition of exclusive breastfeeding - Signs of insufficient breast milk - Common breast problems and how to deal with them - Common problems in infants with breastfeeding - Summary of the meeting - Answer the questions | Virtual webinar and group discussion | - PowerPoint slides - Training video - Audio file - Text messages |
| **4** | Father’s responsibilities in the postpartum period and his supportive role in helping mothers adapt to changes | - Review the previous session summary - Increase communication skills and strengthen couples intimacy for emotional support - Increasing the mother's confidence to play the role of mother, giving a sense of respect and value to the mother - Tangible and understandable support, practical, behavioral and material support, accompanying the mother to help adapt to new situations - Managing household chores and helping to increase the mother's rest and relaxation time - Incentive support and cognitive guidance to help obtain information - Accompanying the mother in all stages of raising the baby, strengthening the sense of belonging and emotional bond and attachment - Summary of the meeting - Answer the questions | Virtual webinar and group discussion | - PowerPoint slides - Audio file - Training video - Text messages |
